# 6-Chloro-3-phenethyl-2-thioxo-2,3-di­hydro­quinazolin-4(1*H*)-one

**DOI:** 10.1107/S1600536810010330

**Published:** 2010-03-27

**Authors:** Norhafizah Mohd Hashim, Hasnah Osman, Afidah Abdul Rahim, Chin Sing Yeap, Hoong-Kun Fun

**Affiliations:** a School of Chemical Science, Universiti Sains Malaysia, 11800 USM, Penang, Malaysia; b X-ray Crystallography Unit, School of Physics, Universiti Sains Malaysia, 11800 USM, Penang, Malaysia

## Abstract

The asymmetric unit of the title quinazolinone compound, C_16_H_13_ClN_2_OS, consists of two crystallographically independent mol­ecules, *A* and *B*. The dihedral angles between the quinazoline and benzene rings are 16.88 (6) and 32.34 (6)° for mol­ecules *A* and *B*, respectively. In the crystal structure, mol­ecules *A* and *B* are linked by two bifurcated inter­molecular N—H⋯S and C—H⋯S hydrogen bonds. Pairs of mol­ecules are further linked by C—H⋯O and C—H⋯Cl hydrogen bonds into a chain aligned approximately along [110].

## Related literature

For the preparation and biological testing of quinazolinone derivatives, see: Glasser *et al.* (1971 ▶). For the preparation of the title compound, see: Butler & Partridge (1959 ▶). For the stability of the temperature controller used for the data collection, see: Cosier & Glazer (1986 ▶).
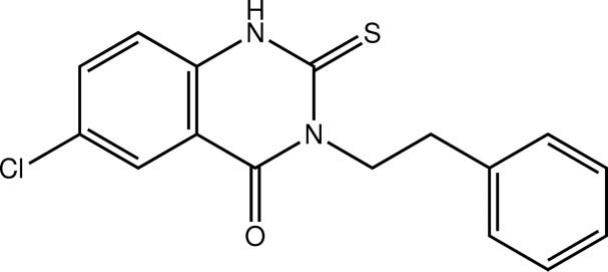


## Experimental

### 

#### Crystal data

C_16_H_13_ClN_2_OS
*M* *_r_* = 316.79
Triclinic, 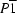

*a* = 9.7228 (4) Å
*b* = 11.8588 (4) Å
*c* = 14.4983 (5) Å
α = 69.709 (1)°
β = 74.395 (1)°
γ = 67.681 (1)°
*V* = 1432.19 (9) Å^3^
*Z* = 4
Mo *K*α radiation
μ = 0.41 mm^−1^
*T* = 100 K
0.45 × 0.19 × 0.07 mm

#### Data collection

Bruker APEX DUO CCD area-detector diffractometer
Absorption correction: multi-scan (*SADABS*; Bruker, 2009 ▶) *T*_min_ = 0.836, *T*_max_ = 0.973
41666 measured reflections
10238 independent reflections
8470 reflections with *I* > 2σ(*I*)
*R*_int_ = 0.032

#### Refinement

*R*[*F* ^2^ > 2σ(*F* ^2^)] = 0.037
*wR*(*F* ^2^) = 0.155
*S* = 1.08
10238 reflections
387 parameters
H atoms treated by a mixture of independent and constrained refinement
Δρ_max_ = 0.80 e Å^−3^
Δρ_min_ = −0.69 e Å^−3^

### 

Data collection: *APEX2* (Bruker, 2009 ▶); cell refinement: *SAINT* (Bruker, 2009 ▶); data reduction: *SAINT*; program(s) used to solve structure: *SHELXTL* (Sheldrick, 2008 ▶); program(s) used to refine structure: *SHELXTL*; molecular graphics: *SHELXTL*; software used to prepare material for publication: *SHELXTL* and *PLATON* (Spek, 2009 ▶).

## Supplementary Material

Crystal structure: contains datablocks global, I. DOI: 10.1107/S1600536810010330/tk2645sup1.cif

Structure factors: contains datablocks I. DOI: 10.1107/S1600536810010330/tk2645Isup2.hkl

Additional supplementary materials:  crystallographic information; 3D view; checkCIF report

## Figures and Tables

**Table 1:** Hydrogen-bond geometry (Å, °)

*D*—H⋯*A*	*D*—H	H⋯*A*	*D*⋯*A*	*D*—H⋯*A*
N2*A*—H2*NA*⋯S1*B*^i^	0.81 (2)	2.53 (2)	3.3362 (12)	172 (2)
N2*B*—H2*NB*⋯S1*A*^ii^	0.91 (2)	2.41 (2)	3.3038 (12)	167.8 (19)
C3*A*—H3*AA*⋯S1*B*^i^	0.93	2.95	3.7207 (13)	142
C3*B*—H3*BA*⋯S1*A*^ii^	0.93	2.87	3.6470 (15)	142
C6*A*—H6*AA*⋯O1*A*^iii^	0.93	2.41	3.2873 (17)	156
C6*B*—H6*BA*⋯O1*B*^iv^	0.93	2.44	3.2810 (18)	151
C16*A*—H16*A*⋯Cl1*A*^iii^	0.93	2.82	3.4630 (15)	127
C16*B*—H16*B*⋯Cl1*B*^iv^	0.93	2.85	3.5836 (13)	137

Symmetry codes: (i) 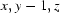; (ii) 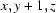; (iii) 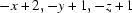; (iv) 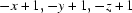.

**Fig. 1.**
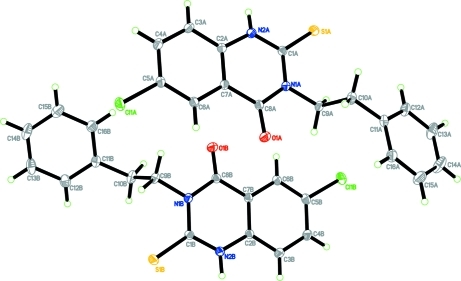
The molecular structure of (I) with displacement ellipsoids at the 50% probability level for non-H atoms.

**Fig. 2.**
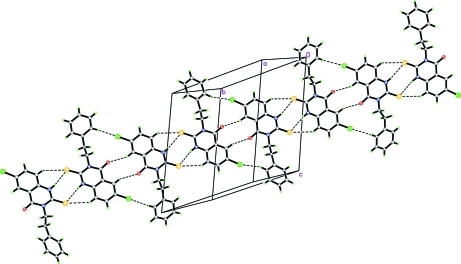
The crystal packing of (I), showing the molecules linked into a one-dimensional chain. Intermolecular hydrogen bonds are shown as dashed lines.

## supplementary crystallographic information

### Comment

Quinazolinones are versatile compounds showing different biological and pharmacological activities. For example, 6-chloro-3-phenyl-2-thioxo-2,3-dihydroquinazolin-4(1*H*)-one has been reported to possess anti-convulsant activity, and a related compound was reported to inhibit maximal electroshock and chemoshock seizures in mice (Glasser *et al.*, 1971).

The asymmetric unit of the title quinazolinone compound, (I), consists of two crystallographically independent molecules, *A* & *B* (Fig. 1). The quinazoline rings are essentially planar with maximum derivations of 0.034 (1) Å for atom N1A, and 0.049 (1) Å for atom C8B. The dihedral angles between the quinazoline and benzene rings are 16.88 (6) and 32.34 (6)° for molecules *A* and *B*, respectively.

In the crystal structure, molecule *A* and *B* are linked together by two bifurcated intermolecular N–H···S and C–H···S hydrogen bonds, Table 1. This pair of molecules is further linked by intermolecular C6A–H6AA···O1A, C16A–H16A···Cl1A, C6B–H6BA···O1B and C16B–H16B···Cl1B hydrogen bonds into a one-dimensional chain (Fig. 2, Table 1).

### Experimental

The title compound (I) was synthesized according to a modification of the method of Butler & Partridge (1959). Equimolar amounts of 2-amino-5-chlorobenzoic acid and 2-phenylethyl isothiocyanate in acetic acid (6 ml) were mixed and stirred under reflux at 423 K for 90 min. The solid that formed was the pure thiol (yield 76.7 %) which produced colourless crystals upon recrystallization from 99.5% ethanol.

### Refinement

The H2NA and H2NB atoms were located from difference Fourier map and refined freely. The remaining H atoms were positioned geometrically and refined using a riding model, with C–H = 0.93 or 0.97 Å. and with *U*_iso_(H) = 1.2 *U*_eq_(C).

### Crystal data

**Table:**

C_16_H_13_ClN_2_OS	*Z* = 4
*M**_r_* = 316.79	*F*(000) = 656
Triclinic, *P*1	*D*_x_ = 1.469 Mg m^−^^3^
Hall symbol: -P 1	Mo *K*α radiation, λ = 0.71073 Å
*a* = 9.7228 (4) Å	Cell parameters from 9954 reflections
*b* = 11.8588 (4) Å	θ = 3.5–37.5°
*c* = 14.4983 (5) Å	µ = 0.41 mm^−^^1^
α = 69.709 (1)°	*T* = 100 K
β = 74.395 (1)°	Plate, colourless
γ = 67.681 (1)°	0.45 × 0.19 × 0.07 mm
*V* = 1432.19 (9) Å^3^	

### Data collection

**Table:**

Bruker APEX DUO CCD area-detector diffractometer	10238 independent reflections
Radiation source: fine-focus sealed tube	8470 reflections with *I* > 2σ(*I*)
graphite	*R*_int_ = 0.032
φ and ω scans	θ_max_ = 32.5°, θ_min_ = 3.7°
Absorption correction: multi-scan (*SADABS*; Bruker, 2009)	*h* = −14→14
*T*_min_ = 0.836, *T*_max_ = 0.973	*k* = −17→17
41666 measured reflections	*l* = −21→21

### Refinement

**Table:**

Refinement on *F*^2^	Primary atom site location: structure-invariant direct methods
Least-squares matrix: full	Secondary atom site location: difference Fourier map
*R*[*F*^2^ > 2σ(*F*^2^)] = 0.037	Hydrogen site location: inferred from neighbouring sites
*wR*(*F*^2^) = 0.155	H atoms treated by a mixture of independent and constrained refinement
*S* = 1.08	*w* = 1/[σ^2^(*F*_o_^2^) + (0.1002*P*)^2^ + 0.3697*P*] where *P* = (*F*_o_^2^ + 2*F*_c_^2^)/3
10238 reflections	(Δ/σ)_max_ = 0.002
387 parameters	Δρ_max_ = 0.80 e Å^−^^3^
0 restraints	Δρ_min_ = −0.69 e Å^−^^3^

### Special details

**Table:**

Experimental. The crystal was placed in the cold stream of an Oxford Cryosystems Cobra open-flow nitrogen cryostat (Cosier & Glazer, 1986) operating at 100.0 (1) K.
Geometry. All esds (except the esd in the dihedral angle between two l.s. planes) are estimated using the full covariance matrix. The cell esds are taken into account individually in the estimation of esds in distances, angles and torsion angles; correlations between esds in cell parameters are only used when they are defined by crystal symmetry. An approximate (isotropic) treatment of cell esds is used for estimating esds involving l.s. planes.
Refinement. Refinement of *F*^2^ against ALL reflections. The weighted *R*-factor wR and goodness of fit *S* are based on *F*^2^, conventional *R*-factors *R* are based on *F*, with *F* set to zero for negative *F*^2^. The threshold expression of *F*^2^ > σ(*F*^2^) is used only for calculating *R*-factors(gt) etc. and is not relevant to the choice of reflections for refinement. *R*-factors based on *F*^2^ are statistically about twice as large as those based on *F*, and *R*- factors based on ALL data will be even larger.

### Fractional atomic coordinates and isotropic or equivalent isotropic displacement parameters (Å^2^)

**Table:**

	*x*	*y*	*z*	*U*_iso_*/*U*_eq_	
Cl1A	1.21736 (4)	0.45726 (3)	0.22883 (2)	0.01818 (8)	
S1A	0.64010 (4)	0.06700 (3)	0.62771 (2)	0.01601 (8)	
O1A	0.86524 (11)	0.39664 (9)	0.58596 (7)	0.01734 (18)	
N1A	0.76021 (12)	0.24848 (10)	0.59831 (8)	0.01229 (18)	
N2A	0.83523 (12)	0.14265 (10)	0.47568 (8)	0.01408 (19)	
C1A	0.75050 (13)	0.15750 (11)	0.56400 (9)	0.0123 (2)	
C2A	0.92745 (14)	0.21423 (11)	0.41665 (9)	0.0128 (2)	
C3A	1.00745 (15)	0.19578 (12)	0.32437 (9)	0.0164 (2)	
H3AA	1.0006	0.1340	0.3018	0.020*	
C4A	1.09685 (15)	0.27023 (12)	0.26702 (9)	0.0164 (2)	
H4AA	1.1502	0.2589	0.2054	0.020*	
C5A	1.10681 (14)	0.36297 (12)	0.30205 (9)	0.0141 (2)	
C6A	1.03064 (14)	0.38072 (11)	0.39387 (9)	0.0133 (2)	
H6AA	1.0401	0.4409	0.4171	0.016*	
C7A	0.93900 (13)	0.30616 (11)	0.45095 (9)	0.01200 (19)	
C8A	0.85589 (13)	0.32317 (11)	0.54824 (9)	0.0125 (2)	
C9A	0.67265 (14)	0.26733 (12)	0.69550 (9)	0.0138 (2)	
H9AA	0.5740	0.2601	0.7028	0.017*	
H9AB	0.6586	0.3522	0.6970	0.017*	
C10A	0.75055 (14)	0.17075 (12)	0.78315 (9)	0.0169 (2)	
H10A	0.7604	0.0857	0.7844	0.020*	
H10B	0.8506	0.1755	0.7756	0.020*	
C11A	0.65870 (14)	0.19892 (12)	0.87884 (9)	0.0150 (2)	
C12A	0.53451 (15)	0.15680 (14)	0.92461 (9)	0.0191 (2)	
H12A	0.5123	0.1056	0.8983	0.023*	
C13A	0.44334 (17)	0.19063 (17)	1.00936 (11)	0.0267 (3)	
H13A	0.3607	0.1622	1.0393	0.032*	
C14A	0.47607 (19)	0.26694 (18)	1.04905 (12)	0.0303 (3)	
H14A	0.4146	0.2906	1.1051	0.036*	
C15A	0.60068 (19)	0.30787 (16)	1.00488 (11)	0.0269 (3)	
H15A	0.6230	0.3586	1.0316	0.032*	
C16A	0.69220 (16)	0.27324 (13)	0.92078 (10)	0.0199 (2)	
H16A	0.7765	0.2998	0.8922	0.024*	
Cl1B	0.31242 (4)	0.52954 (3)	0.78230 (3)	0.02325 (9)	
S1B	0.85424 (4)	0.93740 (3)	0.35950 (2)	0.01609 (8)	
O1B	0.65436 (11)	0.58631 (9)	0.41816 (7)	0.01808 (18)	
N1B	0.74945 (12)	0.74343 (10)	0.39853 (8)	0.01263 (18)	
N2B	0.66378 (12)	0.86059 (10)	0.51420 (8)	0.01375 (19)	
C1B	0.75060 (13)	0.84213 (11)	0.42740 (9)	0.0122 (2)	
C2B	0.57691 (14)	0.78576 (11)	0.57675 (9)	0.0128 (2)	
C3B	0.49338 (15)	0.81019 (12)	0.66673 (9)	0.0162 (2)	
H3BA	0.4937	0.8780	0.6849	0.019*	
C4B	0.41030 (15)	0.73193 (13)	0.72824 (10)	0.0170 (2)	
H4BA	0.3545	0.7469	0.7883	0.020*	
C5B	0.41017 (14)	0.63046 (12)	0.70021 (9)	0.0158 (2)	
C6B	0.48832 (14)	0.60771 (12)	0.60999 (9)	0.0144 (2)	
H6BA	0.4846	0.5418	0.5909	0.017*	
C7B	0.57308 (13)	0.68628 (11)	0.54816 (9)	0.0127 (2)	
C8B	0.65788 (14)	0.66541 (11)	0.45237 (9)	0.0131 (2)	
C9B	0.83828 (14)	0.72078 (12)	0.30298 (9)	0.0142 (2)	
H9BA	0.8687	0.6312	0.3080	0.017*	
H9BB	0.9287	0.7440	0.2893	0.017*	
C10B	0.74844 (14)	0.79720 (13)	0.21685 (9)	0.0186 (2)	
H10C	0.6655	0.7659	0.2260	0.022*	
H10D	0.7064	0.8854	0.2176	0.022*	
C11B	0.84466 (14)	0.78834 (12)	0.11768 (9)	0.0156 (2)	
C12B	0.86257 (16)	0.89791 (13)	0.04431 (10)	0.0201 (2)	
H12B	0.8146	0.9767	0.0569	0.024*	
C13B	0.95240 (17)	0.88930 (15)	−0.04784 (10)	0.0245 (3)	
H13B	0.9635	0.9625	−0.0962	0.029*	
C14B	1.02463 (16)	0.77295 (16)	−0.06746 (10)	0.0247 (3)	
H14B	1.0840	0.7679	−0.1289	0.030*	
C15B	1.00853 (17)	0.66309 (15)	0.00491 (11)	0.0237 (3)	
H15B	1.0576	0.5846	−0.0080	0.028*	
C16B	0.91888 (15)	0.67108 (13)	0.09662 (10)	0.0193 (2)	
H16B	0.9081	0.5975	0.1446	0.023*	
H2NA	0.837 (3)	0.088 (2)	0.4533 (17)	0.028 (5)*	
H2NB	0.657 (2)	0.926 (2)	0.5359 (16)	0.026 (5)*	

### Atomic displacement parameters (Å^2^)

**Table:**

	*U*^11^	*U*^22^	*U*^33^	*U*^12^	*U*^13^	*U*^23^
Cl1A	0.01817 (14)	0.01885 (15)	0.01601 (14)	−0.00978 (11)	0.00160 (10)	−0.00191 (11)
S1A	0.01708 (15)	0.01636 (15)	0.01681 (15)	−0.00897 (11)	0.00014 (11)	−0.00526 (11)
O1A	0.0215 (4)	0.0191 (4)	0.0149 (4)	−0.0093 (3)	−0.0004 (3)	−0.0076 (3)
N1A	0.0133 (4)	0.0127 (4)	0.0107 (4)	−0.0052 (3)	−0.0003 (3)	−0.0031 (3)
N2A	0.0159 (5)	0.0156 (5)	0.0134 (4)	−0.0087 (4)	0.0010 (3)	−0.0055 (4)
C1A	0.0118 (5)	0.0123 (5)	0.0123 (5)	−0.0038 (4)	−0.0014 (4)	−0.0032 (4)
C2A	0.0138 (5)	0.0128 (5)	0.0121 (5)	−0.0049 (4)	−0.0016 (4)	−0.0034 (4)
C3A	0.0197 (5)	0.0181 (5)	0.0137 (5)	−0.0081 (4)	0.0010 (4)	−0.0074 (4)
C4A	0.0176 (5)	0.0189 (6)	0.0128 (5)	−0.0069 (4)	0.0011 (4)	−0.0060 (4)
C5A	0.0137 (5)	0.0147 (5)	0.0124 (5)	−0.0057 (4)	−0.0004 (4)	−0.0021 (4)
C6A	0.0141 (5)	0.0132 (5)	0.0125 (5)	−0.0055 (4)	−0.0019 (4)	−0.0026 (4)
C7A	0.0127 (5)	0.0127 (5)	0.0106 (5)	−0.0047 (4)	−0.0017 (4)	−0.0025 (4)
C8A	0.0129 (5)	0.0128 (5)	0.0114 (5)	−0.0052 (4)	−0.0011 (4)	−0.0024 (4)
C9A	0.0136 (5)	0.0153 (5)	0.0103 (5)	−0.0034 (4)	−0.0001 (4)	−0.0036 (4)
C10A	0.0153 (5)	0.0190 (5)	0.0121 (5)	−0.0022 (4)	−0.0022 (4)	−0.0028 (4)
C11A	0.0147 (5)	0.0173 (5)	0.0109 (5)	−0.0051 (4)	−0.0024 (4)	−0.0010 (4)
C12A	0.0175 (5)	0.0283 (6)	0.0137 (5)	−0.0114 (5)	−0.0008 (4)	−0.0052 (5)
C13A	0.0202 (6)	0.0463 (9)	0.0165 (6)	−0.0165 (6)	0.0027 (5)	−0.0098 (6)
C14A	0.0258 (7)	0.0497 (10)	0.0191 (6)	−0.0130 (7)	0.0031 (5)	−0.0177 (7)
C15A	0.0323 (8)	0.0348 (8)	0.0204 (6)	−0.0142 (6)	−0.0023 (5)	−0.0131 (6)
C16A	0.0235 (6)	0.0235 (6)	0.0151 (5)	−0.0121 (5)	−0.0018 (4)	−0.0038 (5)
Cl1B	0.02524 (17)	0.02368 (17)	0.02023 (16)	−0.01530 (13)	0.00561 (12)	−0.00389 (12)
S1B	0.01661 (15)	0.01511 (15)	0.01741 (15)	−0.00805 (11)	0.00129 (11)	−0.00515 (11)
O1B	0.0222 (5)	0.0191 (4)	0.0170 (4)	−0.0099 (4)	−0.0004 (3)	−0.0081 (3)
N1B	0.0131 (4)	0.0139 (4)	0.0110 (4)	−0.0050 (3)	−0.0002 (3)	−0.0039 (3)
N2B	0.0143 (4)	0.0140 (4)	0.0139 (4)	−0.0068 (4)	0.0010 (3)	−0.0050 (4)
C1B	0.0111 (5)	0.0117 (5)	0.0137 (5)	−0.0038 (4)	−0.0015 (4)	−0.0032 (4)
C2B	0.0127 (5)	0.0130 (5)	0.0124 (5)	−0.0049 (4)	−0.0009 (4)	−0.0029 (4)
C3B	0.0169 (5)	0.0170 (5)	0.0150 (5)	−0.0058 (4)	0.0012 (4)	−0.0072 (4)
C4B	0.0163 (5)	0.0198 (6)	0.0145 (5)	−0.0070 (4)	0.0018 (4)	−0.0061 (4)
C5B	0.0148 (5)	0.0161 (5)	0.0149 (5)	−0.0064 (4)	−0.0002 (4)	−0.0022 (4)
C6B	0.0147 (5)	0.0146 (5)	0.0141 (5)	−0.0066 (4)	−0.0009 (4)	−0.0028 (4)
C7B	0.0126 (5)	0.0138 (5)	0.0117 (5)	−0.0051 (4)	−0.0014 (4)	−0.0031 (4)
C8B	0.0131 (5)	0.0133 (5)	0.0130 (5)	−0.0048 (4)	−0.0018 (4)	−0.0033 (4)
C9B	0.0128 (5)	0.0167 (5)	0.0118 (5)	−0.0035 (4)	−0.0004 (4)	−0.0049 (4)
C10B	0.0135 (5)	0.0263 (6)	0.0122 (5)	−0.0036 (4)	−0.0017 (4)	−0.0042 (4)
C11B	0.0133 (5)	0.0211 (6)	0.0129 (5)	−0.0064 (4)	−0.0027 (4)	−0.0038 (4)
C12B	0.0204 (6)	0.0205 (6)	0.0164 (6)	−0.0059 (5)	−0.0053 (4)	−0.0003 (4)
C13B	0.0231 (6)	0.0330 (7)	0.0138 (6)	−0.0125 (5)	−0.0045 (5)	0.0033 (5)
C14B	0.0186 (6)	0.0439 (9)	0.0132 (5)	−0.0121 (6)	−0.0004 (4)	−0.0088 (5)
C15B	0.0206 (6)	0.0330 (7)	0.0232 (6)	−0.0105 (5)	0.0008 (5)	−0.0153 (6)
C16B	0.0185 (6)	0.0228 (6)	0.0193 (6)	−0.0101 (5)	−0.0002 (4)	−0.0070 (5)

### Geometric parameters (Å, °)

**Table:**

Cl1A—C5A	1.7344 (12)	Cl1B—C5B	1.7376 (13)
S1A—C1A	1.6766 (12)	S1B—C1B	1.6794 (12)
O1A—C8A	1.2181 (14)	O1B—C8B	1.2179 (14)
N1A—C1A	1.3767 (15)	N1B—C1B	1.3775 (15)
N1A—C8A	1.4118 (15)	N1B—C8B	1.4173 (16)
N1A—C9A	1.4795 (15)	N1B—C9B	1.4749 (15)
N2A—C1A	1.3521 (15)	N2B—C1B	1.3503 (15)
N2A—C2A	1.3850 (15)	N2B—C2B	1.3865 (15)
N2A—H2NA	0.81 (2)	N2B—H2NB	0.91 (2)
C2A—C7A	1.3955 (17)	C2B—C7B	1.3948 (17)
C2A—C3A	1.3988 (16)	C2B—C3B	1.3995 (16)
C3A—C4A	1.3825 (18)	C3B—C4B	1.3846 (18)
C3A—H3AA	0.9300	C3B—H3BA	0.9300
C4A—C5A	1.4042 (17)	C4B—C5B	1.3978 (18)
C4A—H4AA	0.9300	C4B—H4BA	0.9300
C5A—C6A	1.3821 (16)	C5B—C6B	1.3840 (17)
C6A—C7A	1.3981 (16)	C6B—C7B	1.3991 (17)
C6A—H6AA	0.9300	C6B—H6BA	0.9300
C7A—C8A	1.4641 (16)	C7B—C8B	1.4619 (16)
C9A—C10A	1.5329 (17)	C9B—C10B	1.5311 (18)
C9A—H9AA	0.9700	C9B—H9BA	0.9700
C9A—H9AB	0.9700	C9B—H9BB	0.9700
C10A—C11A	1.5040 (17)	C10B—C11B	1.5034 (17)
C10A—H10A	0.9700	C10B—H10C	0.9700
C10A—H10B	0.9700	C10B—H10D	0.9700
C11A—C12A	1.3947 (17)	C11B—C16B	1.4005 (18)
C11A—C16A	1.3961 (18)	C11B—C12B	1.4014 (18)
C12A—C13A	1.3937 (19)	C12B—C13B	1.3991 (19)
C12A—H12A	0.9300	C12B—H12B	0.9300
C13A—C14A	1.389 (2)	C13B—C14B	1.381 (2)
C13A—H13A	0.9300	C13B—H13B	0.9300
C14A—C15A	1.388 (2)	C14B—C15B	1.393 (2)
C14A—H14A	0.9300	C14B—H14B	0.9300
C15A—C16A	1.3904 (19)	C15B—C16B	1.3920 (19)
C15A—H15A	0.9300	C15B—H15B	0.9300
C16A—H16A	0.9300	C16B—H16B	0.9300
			
C1A—N1A—C8A	123.85 (10)	C1B—N1B—C8B	123.62 (10)
C1A—N1A—C9A	119.68 (10)	C1B—N1B—C9B	119.81 (10)
C8A—N1A—C9A	116.40 (9)	C8B—N1B—C9B	116.40 (9)
C1A—N2A—C2A	124.75 (10)	C1B—N2B—C2B	124.63 (10)
C1A—N2A—H2NA	121.4 (16)	C1B—N2B—H2NB	121.2 (14)
C2A—N2A—H2NA	113.9 (16)	C2B—N2B—H2NB	114.2 (14)
N2A—C1A—N1A	117.16 (10)	N2B—C1B—N1B	117.21 (10)
N2A—C1A—S1A	120.41 (9)	N2B—C1B—S1B	120.10 (9)
N1A—C1A—S1A	122.43 (9)	N1B—C1B—S1B	122.68 (9)
N2A—C2A—C7A	118.59 (11)	N2B—C2B—C7B	118.91 (11)
N2A—C2A—C3A	121.15 (10)	N2B—C2B—C3B	120.60 (10)
C7A—C2A—C3A	120.26 (11)	C7B—C2B—C3B	120.49 (11)
C4A—C3A—C2A	119.44 (11)	C4B—C3B—C2B	118.99 (11)
C4A—C3A—H3AA	120.3	C4B—C3B—H3BA	120.5
C2A—C3A—H3AA	120.3	C2B—C3B—H3BA	120.5
C3A—C4A—C5A	119.77 (11)	C3B—C4B—C5B	120.14 (11)
C3A—C4A—H4AA	120.1	C3B—C4B—H4BA	119.9
C5A—C4A—H4AA	120.1	C5B—C4B—H4BA	119.9
C6A—C5A—C4A	121.45 (11)	C6B—C5B—C4B	121.45 (11)
C6A—C5A—Cl1A	119.14 (9)	C6B—C5B—Cl1B	119.79 (10)
C4A—C5A—Cl1A	119.41 (9)	C4B—C5B—Cl1B	118.75 (10)
C5A—C6A—C7A	118.48 (11)	C5B—C6B—C7B	118.36 (11)
C5A—C6A—H6AA	120.8	C5B—C6B—H6BA	120.8
C7A—C6A—H6AA	120.8	C7B—C6B—H6BA	120.8
C2A—C7A—C6A	120.58 (11)	C2B—C7B—C6B	120.51 (11)
C2A—C7A—C8A	119.54 (11)	C2B—C7B—C8B	119.27 (11)
C6A—C7A—C8A	119.88 (10)	C6B—C7B—C8B	120.22 (10)
O1A—C8A—N1A	120.21 (11)	O1B—C8B—N1B	119.77 (11)
O1A—C8A—C7A	123.85 (11)	O1B—C8B—C7B	124.15 (11)
N1A—C8A—C7A	115.94 (10)	N1B—C8B—C7B	116.08 (10)
N1A—C9A—C10A	112.27 (10)	N1B—C9B—C10B	111.69 (10)
N1A—C9A—H9AA	109.2	N1B—C9B—H9BA	109.3
C10A—C9A—H9AA	109.2	C10B—C9B—H9BA	109.3
N1A—C9A—H9AB	109.2	N1B—C9B—H9BB	109.3
C10A—C9A—H9AB	109.2	C10B—C9B—H9BB	109.3
H9AA—C9A—H9AB	107.9	H9BA—C9B—H9BB	107.9
C11A—C10A—C9A	109.30 (10)	C11B—C10B—C9B	111.86 (10)
C11A—C10A—H10A	109.8	C11B—C10B—H10C	109.2
C9A—C10A—H10A	109.8	C9B—C10B—H10C	109.2
C11A—C10A—H10B	109.8	C11B—C10B—H10D	109.2
C9A—C10A—H10B	109.8	C9B—C10B—H10D	109.2
H10A—C10A—H10B	108.3	H10C—C10B—H10D	107.9
C12A—C11A—C16A	118.80 (12)	C16B—C11B—C12B	118.55 (12)
C12A—C11A—C10A	120.82 (11)	C16B—C11B—C10B	121.05 (12)
C16A—C11A—C10A	120.31 (11)	C12B—C11B—C10B	120.40 (12)
C13A—C12A—C11A	120.67 (12)	C13B—C12B—C11B	120.26 (13)
C13A—C12A—H12A	119.7	C13B—C12B—H12B	119.9
C11A—C12A—H12A	119.7	C11B—C12B—H12B	119.9
C14A—C13A—C12A	119.92 (13)	C14B—C13B—C12B	120.46 (13)
C14A—C13A—H13A	120.0	C14B—C13B—H13B	119.8
C12A—C13A—H13A	120.0	C12B—C13B—H13B	119.8
C15A—C14A—C13A	119.85 (13)	C13B—C14B—C15B	119.95 (13)
C15A—C14A—H14A	120.1	C13B—C14B—H14B	120.0
C13A—C14A—H14A	120.1	C15B—C14B—H14B	120.0
C14A—C15A—C16A	120.17 (13)	C16B—C15B—C14B	119.86 (13)
C14A—C15A—H15A	119.9	C16B—C15B—H15B	120.1
C16A—C15A—H15A	119.9	C14B—C15B—H15B	120.1
C15A—C16A—C11A	120.56 (13)	C15B—C16B—C11B	120.92 (13)
C15A—C16A—H16A	119.7	C15B—C16B—H16B	119.5
C11A—C16A—H16A	119.7	C11B—C16B—H16B	119.5
			
C2A—N2A—C1A—N1A	−1.69 (18)	C2B—N2B—C1B—N1B	1.50 (18)
C2A—N2A—C1A—S1A	178.65 (10)	C2B—N2B—C1B—S1B	−178.47 (9)
C8A—N1A—C1A—N2A	−1.94 (17)	C8B—N1B—C1B—N2B	2.93 (17)
C9A—N1A—C1A—N2A	−178.71 (10)	C9B—N1B—C1B—N2B	178.14 (10)
C8A—N1A—C1A—S1A	177.71 (9)	C8B—N1B—C1B—S1B	−177.10 (9)
C9A—N1A—C1A—S1A	0.94 (16)	C9B—N1B—C1B—S1B	−1.90 (16)
C1A—N2A—C2A—C7A	2.14 (19)	C1B—N2B—C2B—C7B	−2.23 (19)
C1A—N2A—C2A—C3A	−177.68 (12)	C1B—N2B—C2B—C3B	178.31 (12)
N2A—C2A—C3A—C4A	179.17 (12)	N2B—C2B—C3B—C4B	−178.73 (12)
C7A—C2A—C3A—C4A	−0.64 (19)	C7B—C2B—C3B—C4B	1.81 (19)
C2A—C3A—C4A—C5A	0.2 (2)	C2B—C3B—C4B—C5B	−0.2 (2)
C3A—C4A—C5A—C6A	1.0 (2)	C3B—C4B—C5B—C6B	−2.0 (2)
C3A—C4A—C5A—Cl1A	−179.18 (10)	C3B—C4B—C5B—Cl1B	176.70 (10)
C4A—C5A—C6A—C7A	−1.73 (18)	C4B—C5B—C6B—C7B	2.37 (19)
Cl1A—C5A—C6A—C7A	178.42 (9)	Cl1B—C5B—C6B—C7B	−176.29 (10)
N2A—C2A—C7A—C6A	−179.95 (11)	N2B—C2B—C7B—C6B	179.12 (11)
C3A—C2A—C7A—C6A	−0.14 (18)	C3B—C2B—C7B—C6B	−1.41 (19)
N2A—C2A—C7A—C8A	0.91 (17)	N2B—C2B—C7B—C8B	−1.31 (17)
C3A—C2A—C7A—C8A	−179.27 (11)	C3B—C2B—C7B—C8B	178.16 (11)
C5A—C6A—C7A—C2A	1.31 (18)	C5B—C6B—C7B—C2B	−0.68 (18)
C5A—C6A—C7A—C8A	−179.56 (11)	C5B—C6B—C7B—C8B	179.76 (11)
C1A—N1A—C8A—O1A	−175.62 (11)	C1B—N1B—C8B—O1B	174.32 (12)
C9A—N1A—C8A—O1A	1.25 (17)	C9B—N1B—C8B—O1B	−1.03 (17)
C1A—N1A—C8A—C7A	4.67 (17)	C1B—N1B—C8B—C7B	−6.12 (17)
C9A—N1A—C8A—C7A	−178.46 (10)	C9B—N1B—C8B—C7B	178.53 (10)
C2A—C7A—C8A—O1A	176.27 (12)	C2B—C7B—C8B—O1B	−175.33 (12)
C6A—C7A—C8A—O1A	−2.87 (18)	C6B—C7B—C8B—O1B	4.24 (19)
C2A—C7A—C8A—N1A	−4.03 (16)	C2B—C7B—C8B—N1B	5.14 (17)
C6A—C7A—C8A—N1A	176.82 (10)	C6B—C7B—C8B—N1B	−175.29 (11)
C1A—N1A—C9A—C10A	81.62 (14)	C1B—N1B—C9B—C10B	−88.18 (13)
C8A—N1A—C9A—C10A	−95.39 (12)	C8B—N1B—C9B—C10B	87.36 (13)
N1A—C9A—C10A—C11A	177.68 (10)	N1B—C9B—C10B—C11B	172.27 (10)
C9A—C10A—C11A—C12A	82.00 (15)	C9B—C10B—C11B—C16B	59.08 (16)
C9A—C10A—C11A—C16A	−94.84 (14)	C9B—C10B—C11B—C12B	−120.54 (13)
C16A—C11A—C12A—C13A	1.5 (2)	C16B—C11B—C12B—C13B	0.26 (19)
C10A—C11A—C12A—C13A	−175.42 (13)	C10B—C11B—C12B—C13B	179.89 (12)
C11A—C12A—C13A—C14A	−0.1 (2)	C11B—C12B—C13B—C14B	−0.2 (2)
C12A—C13A—C14A—C15A	−0.8 (3)	C12B—C13B—C14B—C15B	−0.2 (2)
C13A—C14A—C15A—C16A	0.3 (3)	C13B—C14B—C15B—C16B	0.4 (2)
C14A—C15A—C16A—C11A	1.1 (2)	C14B—C15B—C16B—C11B	−0.3 (2)
C12A—C11A—C16A—C15A	−2.0 (2)	C12B—C11B—C16B—C15B	−0.04 (19)
C10A—C11A—C16A—C15A	174.94 (13)	C10B—C11B—C16B—C15B	−179.66 (12)

### Hydrogen-bond geometry (Å, °)

**Table:**

*D*—H···*A*	*D*—H	H···*A*	*D*···*A*	*D*—H···*A*
N2A—H2NA···S1B^i^	0.81 (2)	2.53 (2)	3.3362 (12)	172 (2)
N2B—H2NB···S1A^ii^	0.91 (2)	2.41 (2)	3.3038 (12)	167.8 (19)
C3A—H3AA···S1B^i^	0.93	2.95	3.7207 (13)	142
C3B—H3BA···S1A^ii^	0.93	2.87	3.6470 (15)	142
C6A—H6AA···O1A^iii^	0.93	2.41	3.2873 (17)	156
C6B—H6BA···O1B^iv^	0.93	2.44	3.2810 (18)	151
C16A—H16A···Cl1A^iii^	0.93	2.82	3.4630 (15)	127
C16B—H16B···Cl1B^iv^	0.93	2.85	3.5836 (13)	137

Symmetry codes: (i) *x*, *y*−1, *z*; (ii) *x*, *y*+1, *z*; (iii) −*x*+2, −*y*+1, −*z*+1; (iv) −*x*+1, −*y*+1, −*z*+1.
